# Exploring the effect of lifestyle behaviors and socioeconomic status on atrial fibrillation: the mediating role of 91 inflammatory cytokines

**DOI:** 10.3389/fcvm.2024.1401384

**Published:** 2024-09-12

**Authors:** Yiheng Liu, Mingsheng Huang, Yue Sun, Weiran Dai

**Affiliations:** ^1^ Department of Cardiology, The Second Affiliated Hospital of Chongqing Medical University, Chongqing, China; ^2^ Department of Neurosurgery, The Second Affiliated Hospital of Chongqing Medical University, Chongqing, China; ^3^ Department of Endocrinology, The First Affiliated Hospital of Chongqing Medical University, Chongqing, China

**Keywords:** atrial fibrillation, Mendelian randomization, inflammatory cytokines, lifestyle, socioeconomic

## Abstract

**Background:**

Atrial fibrillation (AF) is one of the most prevalent cardiac arrhythmias and has a significant economic and social burden. Whether it is associated with lifestyle behaviors and socioeconomic status is currently poorly understood. This study aimed to explore the relationship among these factors and determine the role of inflammatory cytokines.

**Method:**

We investigated the causal effects of lifestyle behaviors and socioeconomic status on AF using bidirectional two-sample Mendelian randomization (MR). Instrumental variables were obtained from a publicly available genome-wide association study. A two-step MR was conducted to determine the mediating role of 91 inflammatory cytokines. Inverse variance weighted was used as the main method with four supplementary MR methods. To obtain more robust results, several sensitivity analyses were conducted.

**Result:**

The results indicated that seven of the lifestyle behaviors [smoking initiation, vegetable intake, coffee consumption (cups/day), dozing, lifetime smoking index, napping, and alcohol abuse] were potential risk factors for AF. One socioeconomic status, education attainment (years of education), was causally associated with a decreased risk of AF. Moreover, we found that thymic stromal lymphopoietin, CD40l receptor, C-X-C motif chemokine 6, and C-X-C motif chemokine 11 levels mediated the causal effect, at proportions of 13.6%, 4.1%, 4.3%, and 6.9%, respectively.

**Conclusion:**

Our findings provide insight into the relationship between lifestyle behaviors, socioeconomic status, and AF. Inflammatory cytokines are potential mediators of this relationship.

## 1 Introduction

Atrial fibrillation (AF) is a rapid cardiac arrhythmia that is prevalent globally, particularly among the elderly (1, 2). It has been strongly linked to several diseases, including stroke, coronary heart disease (CHD), and heart failure. The disease is a major cause of all-cause mortality (3, 4). The risk of AF recurrence is high even after surgical or pharmacological treatment (5). Currently, there are no effective treatments to prevent AF recurrence (6).

Lifestyle factors and socioeconomic status are modifiable determinants that may influence AF risk and management. Previous research has demonstrated their association with reduced all-cause mortality (7) and other cardiovascular outcomes, including coronary artery disease, heart failure, and sudden cardiac death (8). Although these studies establish associations, they cannot determine causality. Consequently, investigating the underlying causal relationships between these factors and AF is essential for developing effective prevention and management strategies. The mechanisms of AF are not well understood and need to be explored (9). Of note, inflammatory cytokines, such as C-reactive protein (CRP) and interleukins, are known to influence the severity of AF (10, 11). Given that the development of AF is closely associated with changes in inflammatory cytokines (12, 13), it is imperative to explore the mediating role of inflammatory cytokines in the relationship between lifestyle behaviors, socioeconomic status, and AF.

Mendelian randomization (MR) is a powerful tool that utilizes genetic variants allocated randomly at birth to evaluate causal relationships. The findings detected by MR are less prone to reverse causation and confounding biases. The results of MR often mirror those of randomized controlled trials (RCTs) (14, 15). In this study, we aimed to analyze the causal relationship between lifestyle behavior, socioeconomic status, and AF using MR methods. Modifying lifestyle factors and socioeconomic conditions could effectively reduce the incidence of AF, thereby alleviating the associated financial and medical burdens. To elucidate the underlying mechanisms, this study explored the potential mediating role of 91 inflammatory cytokines in the development of AF.

## 2 Materials and methods

### 2.1 Study design

**Figure 1:**
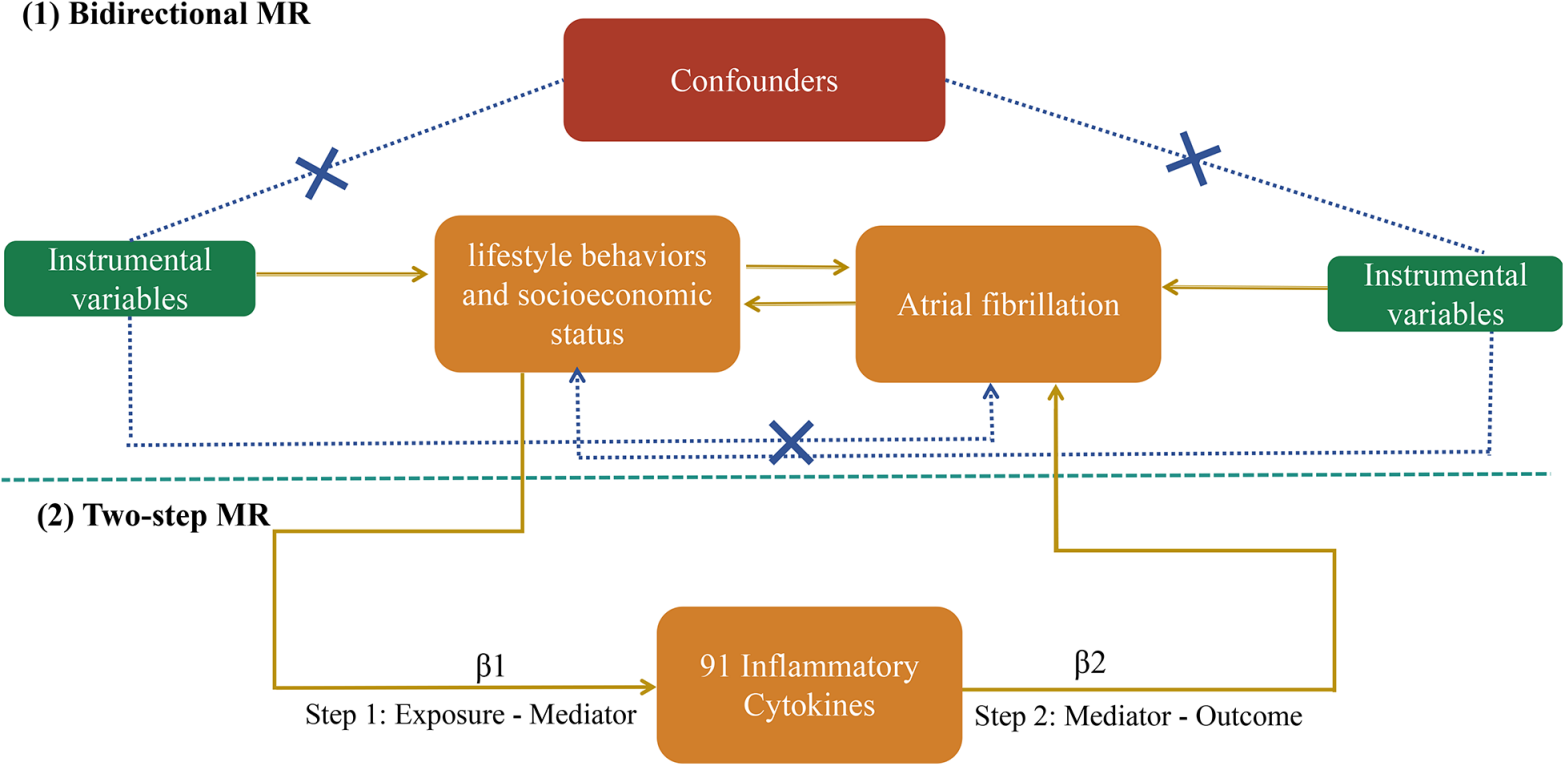
Summary of MR assumptions and the study's flow diagram. MR, Mendelian randomization.

In general, MR analysis should meet three fundamental assumptions: (1) instrumental variables (IVs) should be closely associated with the exposure; (2) the IVs should not be linked to any confounding factors; and (3) the IVs may contribute to the outcome only by influencing the risk factors (Figure 1). The entire MR analysis was performed using the TwoSampleMR package (version 0.5.7) and MR-PRESSO (version 1.0) in R Version (4.2.1) (16).

This study employed a meticulously designed bidirectional MR analysis to investigate the causal relationship between lifestyle factors, socioeconomic status, and AF. We examined the impact of these factors on AF risk and explored potential reverse causality by treating AF as an exposure. To avoid the influence of heterogeneity on the estimated causal effects, random effect inverse variance weighted (RE-IVW) was applied as the primary outcome. In addition, four other analytical methods (MR Egger, weighted median, simple mode, and weighted mode) were adopted to obtain more robust results. Several sensitivity analyses were performed to assess the results and identify potential confounders.

A two-step MR analysis was performed to identify the mediating role of inflammatory cytokines in the relationship between the exposures affecting AF. We first explored the causal effect of exposure on inflammatory factors (β1).

Qualified exposures were defined as risk factors influencing AF without being affected by AF itself, based on bidirectional Mendelian randomization analysis. Subsequently, we examined the influence of the selected mediator on AF (β2). The mediation proportion, representing the indirect effect relative to the total effect, was calculated as described previously (17). Figure 1 outlines the study design.

### 2.2 The data source of the included genome-wide association study

To ensure data consistency and reduce heterogeneity, our analysis primarily focused on genome-wide association study (GWAS) data from individuals of European ancestry, given their greater availability and quality. We obtained single nucleotide polymorphisms (SNPs) from publicly accessible GWAS studies and extracted summary statistics for 91 circulating inflammatory cytokines from a meta-analysis involving 14,824 participants across 11 European cohorts (18). The summary level data used in this study were obtained from https://www.phpc.cam.ac.uk/ceu/proteins and the GWAS Catalog database (accession numbers GCST90274758 to GCST90274848). The dataset used for AF was from the FinnGen consortium (R9) comprising 50,743 cases and 210,652 controls (19). All the 261,395 participants were of European ancestry. The SNPs associated with smoking initiation and alcohol consumption were derived from a GWAS and Sequencing Consortium of Alcohol and Nicotine (GSCAN) use (20), a study that investigated the effect of alcohol use and smoking initiation from a genetic perspective. Genetic associations for dozing, getting up, insomnia, morningness, napping, sensitivity to environmental stress and adversity (SESA), sleep duration, snoring, and the lifetime smoking index were obtained from the UK Biobank (21, 22). We obtained genetic variants associated with physical activity (PA) from a published meta-analysis leveraging data from the UK Biobank, encompassing up to 91,084 participants (23). SNPs linked to coffee consumption were extracted from a recent GWAS involving a maximum of 91,462 participants (24). Summary statistical data for tea intake, vegetable intake, household income, and the Townsend Deprivation Index were retrieved from the Medical Research Council Integrative Epidemiology Unit GWAS database (https://gwas.mrcieu.ac.uk/). It is important to note that this database primarily includes individuals of European ancestry. Summary statistics for alcohol abuse were obtained from the UK Biobank, encompassing data from multiple ancestry groups, with the majority of European descent (25). Summary level data for education attainment (college completion) and education attainment (years of education) were obtained from a GWAS comprising 470,941 participants of European ancestry (26). The detailed information and definition of the above traits are presented in Supplementary Table S1.

### 2.3 Selection of genetic instruments variables

Initially, we extracted SNPs robustly associated with exposure at the genome-wide significance threshold (*p* < 5 × 10^−8^). However, when the number of SNPs was limited, a more lenient threshold for the genome-wide significance level was set at *p* < 5 × 10^−6^, which had been used in previous studies (27). Next, SNP clumping (kb = 10,000, *r*^2^ < 0.001) was conducted using the European sample to avoid the linkage disequilibrium (LD). We ensured consistent effect allele orientation across all datasets and excluded palindromic sequences with ambiguous allele frequencies. We identified and excluded SNPs potentially associated with both the outcome (atrial fibrillation) and outcome-related confounders using the PhenoScanner database tool (http://www.phenoscanner.medschl.cam.ac.uk/). Specific confounders excluded included body mass index, age, smoking status, chronic inflammation, hypertension, coronary artery disease, chronic renal failure, diabetes, and the use of medications such as anticoagulants, antiplatelet agents, and lipid-lowering drugs. To investigate the statistical strength of the variables, we calculated the F-statistic using the following formula: $F\;=\;R^{2}/\left(1-R^{2})\;\times \;(N-k-1\right)/k$ (28). SNPs with F < 10 were filtered to select instruments variables (IVs) with strong power. Detailed information on all the included IVs are presented in Supplementary Table S2–S4.

### 2.4 Statistical analysis

To assess the potential causal relationships between lifestyle behaviors, socioeconomic status, inflammatory cytokines, and AF, we employed a RE-IVW approach. This method mimics the RCT design by leveraging the random assignment principle, increasing statistical power and reliability (29). Nonetheless, the RE-IVW method remains susceptible to bias due to pleiotropy. Four other MR methods (MR Egger, weighted median, simple mode, and weighted mode) were employed to further assess the stability of the results. A statistical significance threshold of *p* < 0.05 was selected to determine the causal relationship.

Several sensitivity analyses were conducted to identify cases of pleiotropy. Cochrane's Q statistic and funnel plots were used to measure heterogeneity among the estimates from each SNP by the IVW and MR Egger methods. Heterogeneity among genetic instruments was assessed using Cochran's Q statistic, with a *p*-value < 0.05 indicating significant heterogeneity. To detect horizontal pleiotropy, we employed the MR-PRESSO global test (30). Directional pleiotropy was evaluated using the MR Egger test, with a *p*-value < 0.05 indicating its presence. Sensitivity analysis using leave-one-out analysis assessed the influence of individual SNPs on the overall results by sequentially excluding each SNP (31). The forest plots and scatter plots were constructed to display the results of the five MR methods.

## 3 Results

### 3.1 Influence of lifestyle behaviors and socioeconomic status on AF (forward MR analysis)

**Figure 2:**
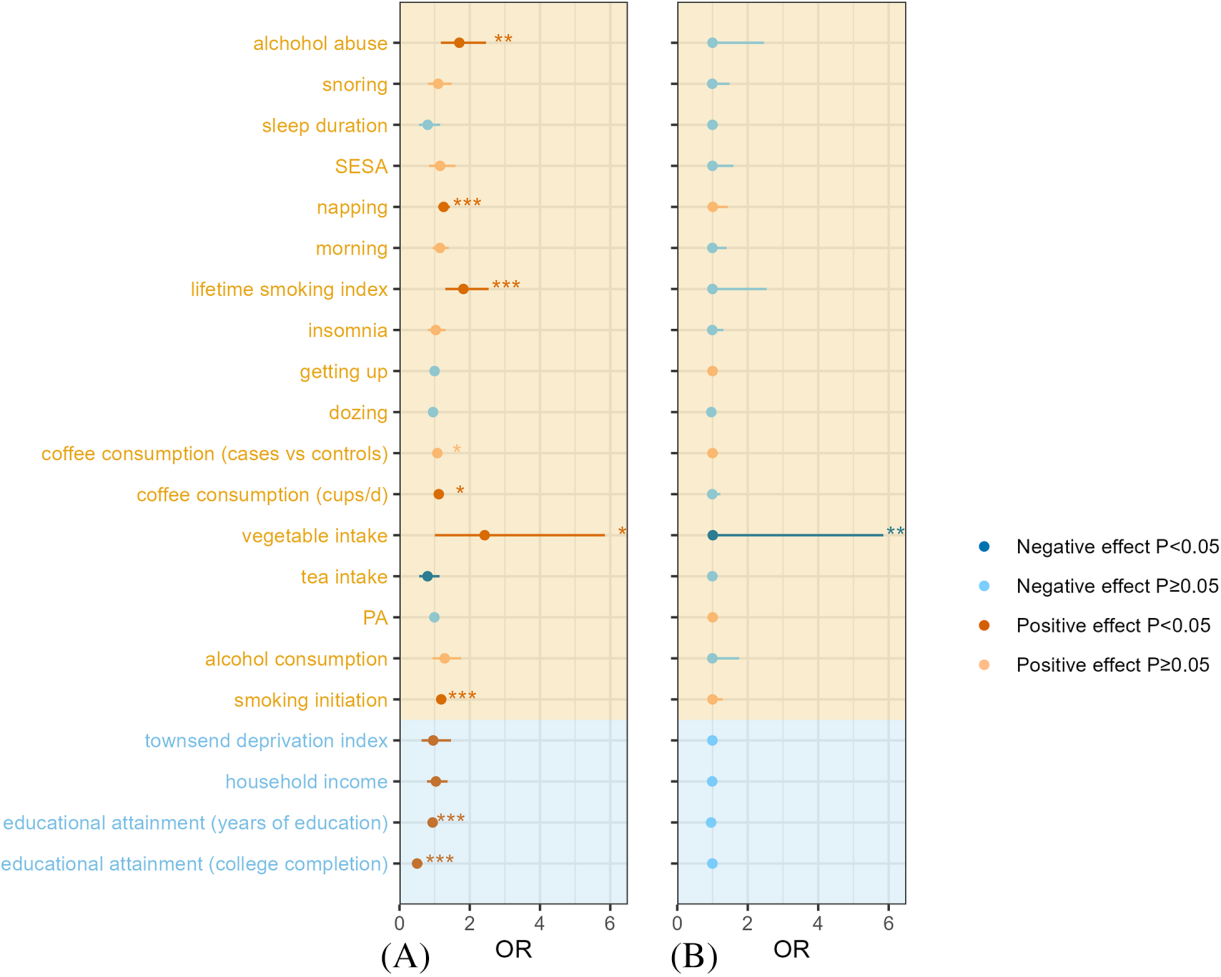
Evaluation of the forward and reverse relationship between lifestyle behaviors, socioeconomic status and AF by using the IVW method: **(A)** Forward MR and **(B)** Reverse MR. AF, atrial fibrillation; IVW, inverse variance weighted; MR, Mendelian randomization; PA, physical activity; SESA, sensitivity to environmental stress and adversity; OR, odds ratio.

In the forward MR analysis, smoking initiation [odds ratio (OR): 1.187, 95% confidence interval (CI): 1.088–1.294, *p* < 0.001], vegetable intake (OR: 2.425, 95% CI: 1.005–5.848, *p* = 0.048), coffee consumption (cups/day) (OR: 1.117, 95% CI: 1.020–1.224, *p* = 0.017), coffee consumption (cases vs. controls) (OR: 1.078, 95% CI: 1.016–1.143, *p* = 0.011), lifetime smoking index (OR: 1.819, 95% CI: 1.304–2.536, *p* < 0.001), napping (OR: 1.256, 95% CI: 1.100–1.433, *p* < 0.001), alcohol abuse (OR: 1.703, 95% CI: 1.178–2.462, *p* = 0.004), education attainment (college completion) (OR: 0.501, 95% CI: 0.383–0.655, *p* < 0.001), and education attainment (years of education) (OR: 0.942, 95% CI: 0.917–0.969, *p* < 0.001) were associated with the risk of AF, as determined using the IVW method. No statistically significant correlations were found between alcohol consumption, physical activity, tea intake, sleep patterns (dozing, getting up, insomnia, morningness, sleep duration, and snoring), socioeconomic status (household income and the Townsend deprivation index), and AF (Table 1, Figure 2).

**Table 1:** Forward MR reveals causal links between lifestyle behaviors, socioeconomic status, and AF incidence by the IVW method.

Exposure	Outcome						
		NSNPs	Beta	SE	*p*	OR	95% CI
Lifestyle							
Smoking initiation	AF	219	0.172	0.044	0.000	1.187	1.088–1.294
Alcohol consumption	AF	33	0.251	0.159	0.113	1.286	0.942–1.754
PA	AF	8	−0.007	0.035	0.849	0.993	0.927–1.064
Tea intake	AF	40	−0.224	0.182	0.218	0.799	0.559–1.141
Vegetable intake	AF	17	0.886	0.449	0.049	2.425	1.005–5.848
Coffee consumption (cups/day)	AF	5	0.111	0.047	0.017	1.118	1.020–1.224
Coffee consumption (cases vs. controls)	AF	4	0.075	0.030	0.012	1.078	1.016–1.143
Dozing	AF	22	−0.045	0.038	0.238	0.956	0.887–1.030
Getting up	AF	25	0.000	0.001	0.889	1.000	0.997–1.001
Insomnia	AF	11	0.033	0.122	0.790	1.033	0.813–1.312
Lifetime smoking index	AF	110	0.598	0.170	0.000	1.819	1.304–2.536
Morningness	AF	91	0.140	0.100	0.161	1.151	0.945–1.400
Napping	AF	6	0.228	0.067	0.001	1.256	1.100–1.433
SESA	AF	39	0.146	0.163	0.371	1.157	0.840–1.594
Sleep duration	AF	39	−0.221	0.187	0.238	0.802	0.555–1.157
Snoring	AF	27	0.095	0.153	0.534	1.100	0.814–1.485
Alcohol abuse	AF	2	0.533	0.188	0.005	1.703	1.178–2.462
Socioeconomic status							
Educational attainment (college completion)	AF	198	−0.691	0.137	0.000	0.501	0.383–0.655
Educational attainment (years of education)	AF	206	−0.059	0.014	0.000	0.943	0.917–0.969
Household income	AF	45	0.036	0.142	0.801	1.036	0.785–1.368
Townsend deprivation index	AF	17	−0.042	0.216	0.847	0.959	0.627–1.465

MR, Mendelian randomization; AF, atrial fibrillation; IVW, inverse variance weighted; PA, physical activity; SESA, sensitivity to environmental stress and adversity; NSNPs, number of single nucleotide polymorphisms; SE, standard error; OR, odds ratio; 95% CI, 95% confidence interval.

Surprisingly, the results obtained by the MR Egger and IVW methods were not aligned perfectly in terms of direction for vegetable intake and educational attainment (college completion). The results of the rest of the tests were stable (Supplementary Table S5). All IVs had an F value exceeding 10 (Supplementary Table S2).

To account for potential heterogeneity, random effects models were used. MR Egger regression identified horizontal pleiotropy in tea intake and educational attainment (college completion) (Egger intercept *p* = 0.019 and 0.003, respectively), rendering these results unreliable. The remaining findings were robust (Egger intercept *p* > 0.05). Additional sensitivity analyses using MR-PRESSO corroborated these results, as detailed in Supplementary Table S6.

### 3.2 The influence of AF on lifestyle behaviors and socioeconomic status (reverse MR analysis)

The results of the reverse MR analysis showing the association of AF on lifestyle behaviors and socioeconomic status are presented in Table 2, Supplementary Table S7, and Figure 2. All the included SNPs met the criteria of an F value greater than 10, calculated using the formula listed above (Supplementary Table S3). Our findings indicated a causal relationship between increased vegetable intake and an increased risk of AF (OR: 1.009, 95% CI: 1.002–1.015, *p* = 0.006). In contrast, no causal association was observed between AF and other lifestyle factors or socioeconomic status (Table 2, Figure 2). Given the discrepancy between the IVW and MR Egger methods for vegetable intake and PA, sensitivity analyses were conducted. Although the overall results were consistent across multiple MR methods (Supplementary Table S7), the MR Egger intercept *p*-values for PA and vegetable intake (0.009 and 0.030, respectively) suggested potential horizontal pleiotropy, necessitating cautious interpretation of these findings.

**Table 2:** Reverse MR reveals causal links between AF and changes in lifestyle behaviors and socioeconomic status.

Outcome	Exposure						
		NSNPs	Beta	SE	*p*	OR	95% CI
Lifestyle							
Smoking initiation	AF	90	0.001	0.008	0.882	1.001	0.985–1.016
Alcohol consumption	AF	89	−0.005	0.004	0.270	0.995	0.986–1.003
PA	AF	92	0.005	0.055	0.928	1.005	0.901–1.119
Tea intake	AF	92	−0.005	0.004	0.201	0.995	0.986–1.002
Vegetable intake	AF	92	0.009	0.003	0.006	1.009	1.002–1.015
Coffee consumption (cups/day)	AF	32	−0.007	0.017	0.690	0.993	0.961–1.026
Coffee consumption (cases vs. controls)	AF	32	0.001	0.032	0.985	1.001	0.939–1.065
Dozing	AF	72	−0.034	0.022	0.123	0.967	0.926–1.009
Getting up	AF	72	0.002	0.004	0.549	1.002	0.995–1.009
Insomnia	AF	72	−0.006	0.008	0.457	0.994	0.979–1.009
Lifetime smoking index	AF	85	−0.001	0.003	0.816	0.999	0.993–1.005
Morning	AF	72	−0.004	0.005	0.446	0.996	0.986–1.006
Napping	AF	72	0.007	0.016	0.643	1.007	0.976–1.039
SESA	AF	72	−0.001	0.005	0.814	0.999	0.988–1.009
Sleep duration	AF	72	−0.001	0.004	0.891	0.999	0.990–1.007
Snoring	AF	72	−0.007	0.013	0.595	0.993	0.968–1.018
Alcohol abuse	AF	75	0.000	0.012	0.985	1.000	0.976–1.023
Socioeconomic status							
Educational attainment (college completion)	AF	92	−0.003	0.002	0.146	0.997	0.992–1.001
Educational attainment (years of education)	AF	92	−0.040	0.022	0.068	0.961	0.920–1.002
Household income	AF	92	−0.009	0.005	0.070	0.991	0.981–1.000
Townsend deprivation index	AF	92	−0.003	0.003	0.370	0.997	0.990–1.003

MR, Mendelian randomization; AF, atrial fibrillation; PA, physical activity; SESA, sensitivity to environmental stress and adversity; NSNPs, number of single nucleotide polymorphisms; SE, standard error; OR, odds ratio; 95% CI, 95% confidence interval.

### 3.3 The mediation effects of inflammatory cytokines on the impact of lifestyle behavior and socioeconomic status on AF (two-step MR)

**Figure 3:**
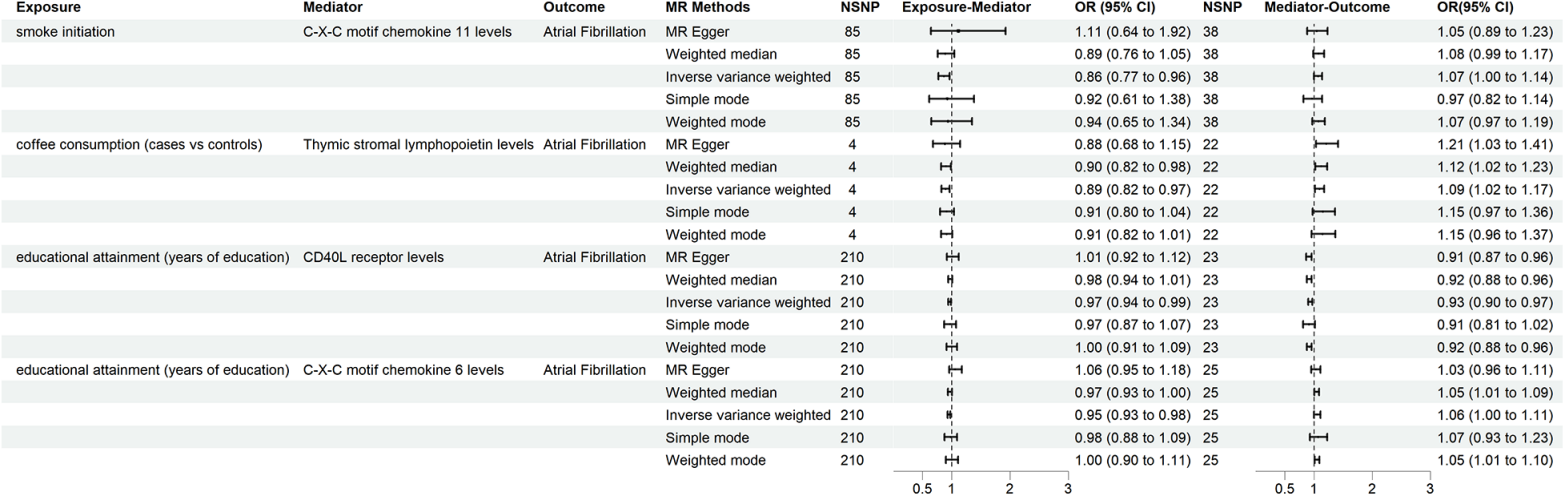
The mediators identified through two-step MR and their relationships with lifestyle behaviors, socioeconomic status, and AF. AF, atrial fibrillation; MR, Mendelian randomization.

**Figure 4:**
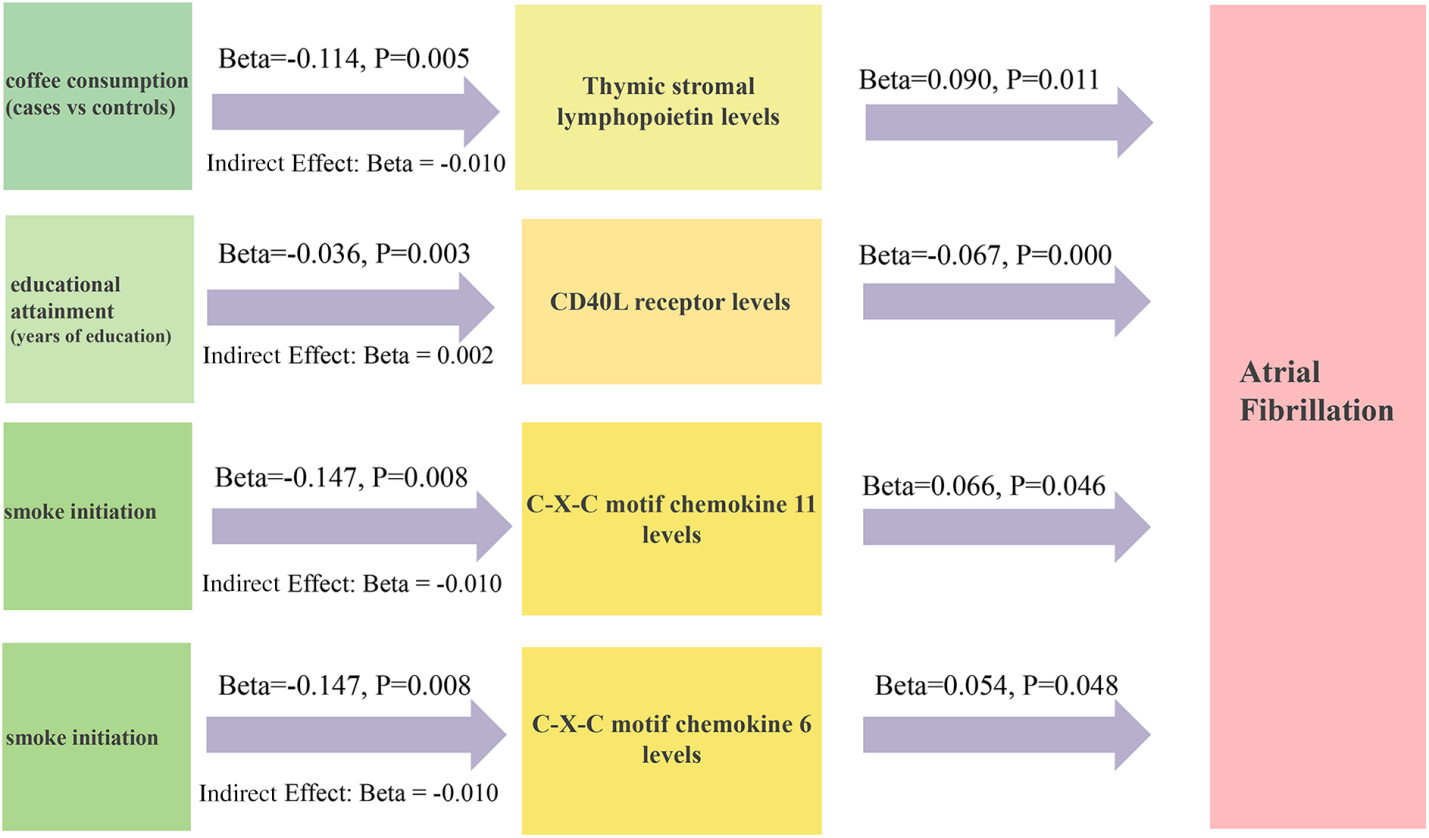
The two-step MR estimates for mediators between lifestyle behaviors, socioeconomic status, and AF. MR, Mendelian randomization; AF, atrial fibrillation.

Forward MR analysis identified smoking initiation, coffee consumption (both cups per day and cases vs. controls), lifetime smoking, napping, alcohol abuse, and educational attainment as causal factors influencing AF risk. We selected SNPs associated with these traits to examine their impact on 91 inflammatory cytokines (Supplementary Table S2). The primary and sensitivity analysis results are presented in Supplementary Table S9. A rigorous selection process narrowed down 91 inflammatory cytokines to four potential mediators influencing the relationship between lifestyle factors, socioeconomic status, and AF: CXCL11, thymic stromal lymphopoietin, CD40l receptor, and CXCL6. Detailed information about the selected genetic instrumental variables, all exhibiting F-statistics greater than 10, is presented in Supplementary Table S4. There was a significant association between smoke and low CXCL11 levels (OR: 0.863, 95% CI: 0.775–0.962, *p* = 0.008). Elevated CXCL11 levels were associated with an increased risk of AF (OR: 1.068, 95% CI: 1.001–1.139, *p* = 0.046), mediating 6.9% of the overall effect. Conversely, coffee consumption was linked to decreased thymic stromal lymphopoietin levels (OR: 0.892, 95% CI: 0.824–0.966, *p* = 0.005) but paradoxically increased AF risk (OR: 1.094, 95% CI: 1.021–1.172, *p* = 0.011), with a mediation proportion of 13.6%. Among the 91 inflammatory cytokines examined, only CD40l receptor and CXCL6 levels were identified as potential mediators between educational attainment and AF risk. For CD40l receptor and CXCL6 levels, education attainment (years of education) was associated with a higher level of inflammatory cytokines (OR: 0.965, 95% CI: 0.943–0.988, *p* = 0.003; and OR: 0.954, 95% CI: 0.931–0.977, *p* = 0.011). Through the IVW method, CD40l receptor levels (OR: 0.935, 95% CI: 0.901–0.970, *p* < 0.001) and CXCL6 (OR: 1.055, 95% CI: 1.001–1.113, *p* = 0.048) exhibited opposite effects on AF. Moreover, the proportion mediated by these two was 4.1% and 4.3%, respectively (Figures 3, 4).

Sensitivity analyses consistently supported the primary findings (Supplementary Table S10). Leave-one-out analysis confirmed the robustness of the causal effects by excluding potentially influential instrumental variables. Symmetrical funnel plots, along with results from additional analyses (Supplementary Figures S1–S4), further corroborated the reliability of our findings.

## 4 Discussion

This study aimed to investigate the bidirectional relationship between lifestyle behaviors, socioeconomic status, and AF. In addition, the mediating role of inflammatory cytokines was investigated. The results indicated that smoking initiation (age at smoking initiation), coffee consumption (cups/day), coffee consumption (cases vs. controls), dozing, lifetime smoking index, napping, and alcohol abuse are associated with an elevated risk of AF. In contrast, educational attainment (years of education) was negatively associated with the development of AF. Two-step MR analysis revealed that the causal relationship of educational attainment (years of education) with the risk of AF was mediated by CD40l receptor levels and CXCL6 (mediation proportions of 4.1% and 4.3%, respectively). Coffee consumption (cases vs. controls) had a casual effect on AF, and this effect was mediated by the level of thymic stromal lymphopoietin (a mediation proportion of 13.6%). When smoke initiation was treated as an exposure, it only affected the incidence of AF by affecting CXCL11 levels, which mediated 9.7% of the causal relationship. These novel findings underscore the significance of targeting the modifiable risk factors, such as lifestyle behaviors and socioeconomic status, as a strategy for preventing the occurrence of AF.

Currently, AF is the most prevalent rapid cardiac arrhythmia (32). Compared with patients without AF, those with AF experience a higher economic burden and poorer quality of life (33, 34). The high recurrence rate of AF underscores the importance of identifying modifiable risk factors. Although traditional observational studies cannot definitively establish causality, MR offers a valuable approach to assess potential causal relationships.

Previous studies have demonstrated that smoke is one of the modifiable risk factors of AF, but evidence for this conclusion is limited (35–37). Studies have indicated that the incidence of AF in active and former smokers is higher than that in patients who never smoked (HR: 1.71, 95% CI: 1.14–2.56; and HR: 1.16, 95% CI: 1.00–1.30, respectively) (38). A recent investigation observed that a higher exposure to secondhand smoking increases the risk of AF compared with non-smokers, with an OR of 3.81 (95% CI 2.02–7.18) (39). Our findings align with previous research regarding the impact of smoking initiation and cumulative smoking exposure on AF. Our results did not support the suggestion by some studies that there was a potential 11% increased risk of venous thromboembolism (VTE) with one to four cups of coffee daily (40). The findings of our study are consistent with those reported by others that coffee consumption (cups/day) and coffee consumption (cases vs. controls) are risk factors of AF. Therefore, we postulate that coffee consumption increases the prevalence of VTE, which in turn elevates the risk of AF. Although drinking at a low concentration is considered to be beneficial for human health, especially in terms of cardiovascular health (41), several other investigations have concluded that alcohol consumption at a high concentration may contribute to the risk of AF (42). Potential mechanisms linking alcohol abuse to AF include an impaired vagal tone, acetaldehyde toxicity, free radical production, and hyperadrenergic states (42). Our findings support alcohol abuse as a risk factor for AF. Further research is needed to explore the association between daytime napping and AF. It has been shown that sleep duration may be an independent risk factor for AF (43). Kanagasabai and Ardern demonstrated that individuals who take midday naps are more likely to develop AF (44). This is consistent with our findings. Specifically, the present results indicated that those who take midday naps are more likely to experience sleeplessness at night. Inadequate sleep will, in turn, trigger inflammation and oxidative stress, potentially contributing to the onset of AF (44). Although previous observational studies have confirmed the relationship between education and health status, particularly in the context of cardiovascular diseases (CVDs), whether there is causality remains to be determined (45, 46). The protective association between higher education and health outcomes may be partially attributed to healthier lifestyle behaviors, such as reduced smoking and increased physical activity (47, 48). In addition, individuals with higher education levels tend to have lower BMIs (49), further contributing to the reduced CVD risk.

There was an accumulation of evidence showing that inflammation has a connection to the occurrence of AF (50). In addition, low-grade systemic inflammation was connected to inflammation in the atrial tissue. For example, Liuba et al. reported that patients with permanent AF had elevated IL-8 levels in the systemic circulation, right atrium, and coronary sinus, and the correlation was high among them (51). The increase in inflammatory cytokines in the circulation causes atrial structural remodeling through collagen turnover (52). Similar to a previous study, TNF-α injection into mice activated myofibroblasts by increasing the secretion of matrix metalloproteinases (MMPs) (53).

The pathophysiology of AF is complex. Previous studies have implicated subclinical inflammation and chronic oxidative stress as key contributors to AF development (54), findings supported by research on CRP (55). CXCL11 and CXCL6, two chemokines involved in inflammation and immune responses, are likely to play pivotal roles in this process. In our study, these two chemokines were found to be associated with the occurrence of AF and their influence was subject to several modifiable risk factors. Similarly, a prospective observational cohort study reported that the C-X-C motif chemokine 12 (CXCL12) level was higher in the AF group than in the control group [3.91 (2.80–10.49) vs. 2.32 (1.57–4.48)] (56). Thymic stromal lymphopoietin, on the other hand, is a cytokine that mainly regulates allergic and inflammatory responses. It is produced by various cell types, including epithelial cells. Although some scholars have demonstrated the association between thymic stromal lymphopoietin and atrial fibrosis, others have shown that thymic stromal lymphopoietin upregulates the expression of CCL2 in primary human lung fibroblasts (57). CD40l, a membrane protein, is an important signaling molecule involved in the activation of T cells. The CD40–CD40l interaction facilitates intercellular communication within the immune system. Soluble CD40l (sCD40l), a cleaved form of CD40l, has been linked to atrial structural alterations in AF patients (58). Given the established role of inflammatory factors in atrial fibrosis, we hypothesize that they may mediate the influence of lifestyle factors and socioeconomic status on AF development. However, further research is required to validate this proposed mechanism.

Despite the strengths of our findings, this study has several limitations that should be acknowledged. First, we verified the effects of lifestyle behaviors and socioeconomic status on AF. Although we included potential mediators to explain the relationship, they only account for part of the relationship, and a greater fraction remains unexplained. Therefore, further studies are advocated to investigate the mechanisms of AF. Second, we did not differentiate between paroxysmal AF, persistent AF, long-standing persistent AF, and permanent AF in GWASs related to AF, which make it difficult to perform a subgroup analysis that might be meaningful. Third, the limited availability of GWAS data on atrial inflammation hindered a comprehensive exploration of the underlying pathophysiological mechanisms. Future studies should address this knowledge gap. Fourth, a potential overlap between exposure and outcome populations may have influenced our findings, necessitating more detailed participant characterization in subsequent research. Finally, the predominantly European focus of the included GWASs limits the generalizability of our results to diverse ethnic populations.

## 5 Conclusion

Our findings strongly suggest that lifestyle factors and socioeconomic status are associated with an increased risk of AF. To effectively reduce AF incidence, public health interventions should target these modifiable determinants. The underlying mechanisms may involve inflammatory cytokine pathways, but further investigations are advocated. Large-scale multi-ethnic randomized controlled trials are needed to corroborate our findings and elucidate the causal relationships.

## Data Availability

The original contributions presented in the study are included in the article/Supplementary Material, further inquiries can be directed to the corresponding author.
