# Supplementary material for: Exploring the effect of lifestyle behaviors and socioeconomic status on atrial fibrillation: the mediating role of 91 inflammatory cytokines
Source: Front Cardiovasc Med. 2024 Sep 12;11:1401384. doi: 10.3389/fcvm.2024.1401384 (PMC11424413; doi:10.3389/fcvm.2024.1401384)
Supplement: Supplementary file 1 [file Datasheet1.docx]

Supplementary Material

# Supplementary Tables

**Table S1.** The source and the detailed information of all the included MR studies. MR, mendelian randomization; AF, Atrial Fibrillation; GSCAN, Global Schizophrenia Collaboration and the Psychiatric Genomics Consortium; GWAS, Genome-Wide Association Study; ID, identity; PA, physical activity; NA, not available; MRC-IE, Medical Research Council Integrative Epidemiology Unit; SESA, Sensitivity to Environmental Stress and Adversity;

**Table S2.** The instrumental variables of lifestyle behaviors and socioeconomic status.AF, Atrial Fibrillation; SNP: Single Nucleotide Polymorphism; SE, Standard Error; EA, Effect Allele; OA, Other Allele; EAF, Effect Allele Frequency; SESA, Sensitivity to Environmental Stress and Adversity; PA, physical activity;

**Table S3.** The instrumental variables of AF. AF, Atrial Fibrillation; SNP: Single Nucleotide Polymorphism; SE, Standard Error; EA, Effect Allele; OA, Other Allele; EAF, Effect Allele Frequency; SESA, Sensitivity to Environmental Stress and Adversity; PA, physical activity;

**Table S4.** The instrumental variables of inflammatory cytokines. AF, Atrial Fibrillation; SNP: Single Nucleotide Polymorphism; SE, Standard Error; EA, Effect Allele; OA, Other Allele; EAF, Effect Allele Frequency;

**Table S5.** Forward MR reveals causal links between lifestyle behaviors, socio-economic status, and AF incidence by the IVW, MR Egger, weighted median, simple mode, weighted mode methods. AF, Atrial Fibrillation; MR, mendelian randomization; IVW, Inverse Variance Weighted; PA, physical activity; SESA, Sensitivity to Environmental Stress and Adversity; OR, Odds Ratio; CI, Confidence Interval; NSNP, Number of Single Nucleotide Polymorphism;

**Table S6.** Sensitivity analysis for the causal results of the forward MR. (lifestyle behaviors and socioeconomic status on AF). MR, mendelian randomization; AF, Atrial Fibrillation; Single Nucleotide Polymorphism; PRESSO, Pleiotropy-Resistant, Egger, and Selection-based Statistic Optimization; PA, physical activity; SESA, Sensitivity to Environmental Stress and Adversity; NA, not available;

**Table S7.** Reverse MR reveals causal links between lifestyle behaviors, socio-economic status, and AF incidence by the IVW, MR Egger, weighted median, simple mode, weighted mode methods. AF, Atrial Fibrillation; MR, mendelian randomization; IVW, Inverse Variance Weighted; PA, physical activity; SESA, Sensitivity to Environmental Stress and Adversity; OR, Odds Ratio; CI, Confidence Interval; NSNP, Number of Single Nucleotide Polymorphism;

**Table S8.** Sensitivity analysis for the causal results of reverse MR (AF on lifestyle behaviors and socioeconomic status). AF, Atrial Fibrillation; MR, mendelian randomization; SNP, Single Nucleotide Polymorphism; PRESSO, Pleiotropy-Resistant, Egger, and Selection-based Statistic Optimization; PA, physical activity; SESA, Sensitivity to Environmental Stress and Adversity; NA, not available;

**Table S9.** The association between mediators and the occurrence of AF using the IVW method and the sensitivity analysis for the tests. AF, Atrial Fibrillation; IVW, Inverse Variance Weighted; NSNP, Number of Single Nucleotide Polymorphism; PRESSO, Pleiotropy-Resistant, Egger, and Selection-based Statistic Optimization; OR, Odds Ratio; CI, Confidence Interval; NA, not available;

**Table S10.** Sensitivity analysis for the causal results of mediators on AF. AF, Atrial Fibrillation; PRESSO, Pleiotropy-Resistant, Egger, and Selection-based Statistic Optimization; SNP, Single Nucleotide Polymorphism; MR, mendelian randomization;

# Supplementary Figures


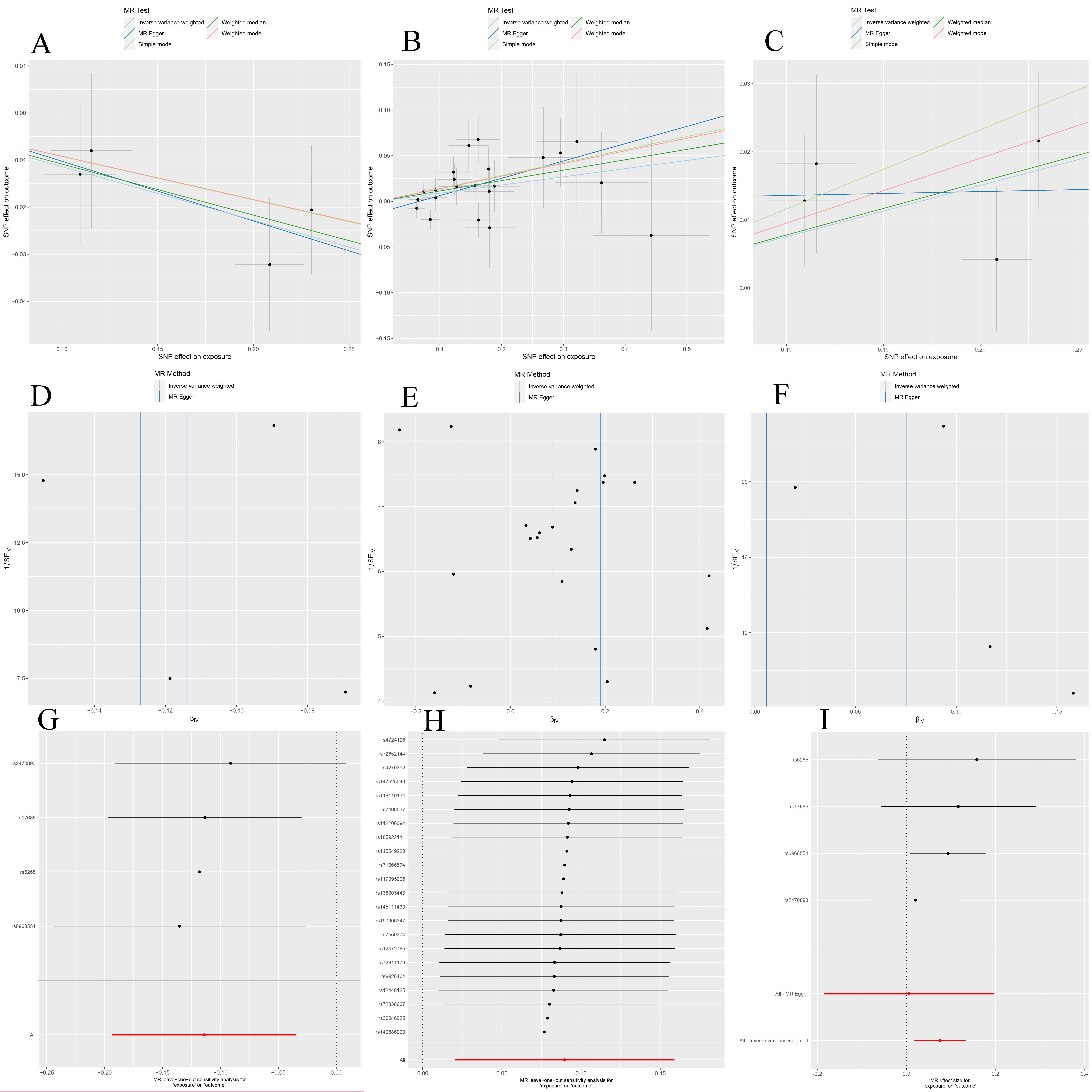


**Figure S1.** Scatter plots, funnel plots and “leave-one-out” results of genetic correlation between coffee consumption (cases vs controls), Thymic stromal lymphopoietin levels, and atrial fibrillation by different MR analysis methods. A; D; G coffee consumption (cases vs controls) on Thymic stromal lymphopoietin levels. B; E; H Thymic stromal lymphopoietin levels on atrial fibrillation. C; F; I coffee consumption (cases vs controls) on atrial fibrillation.


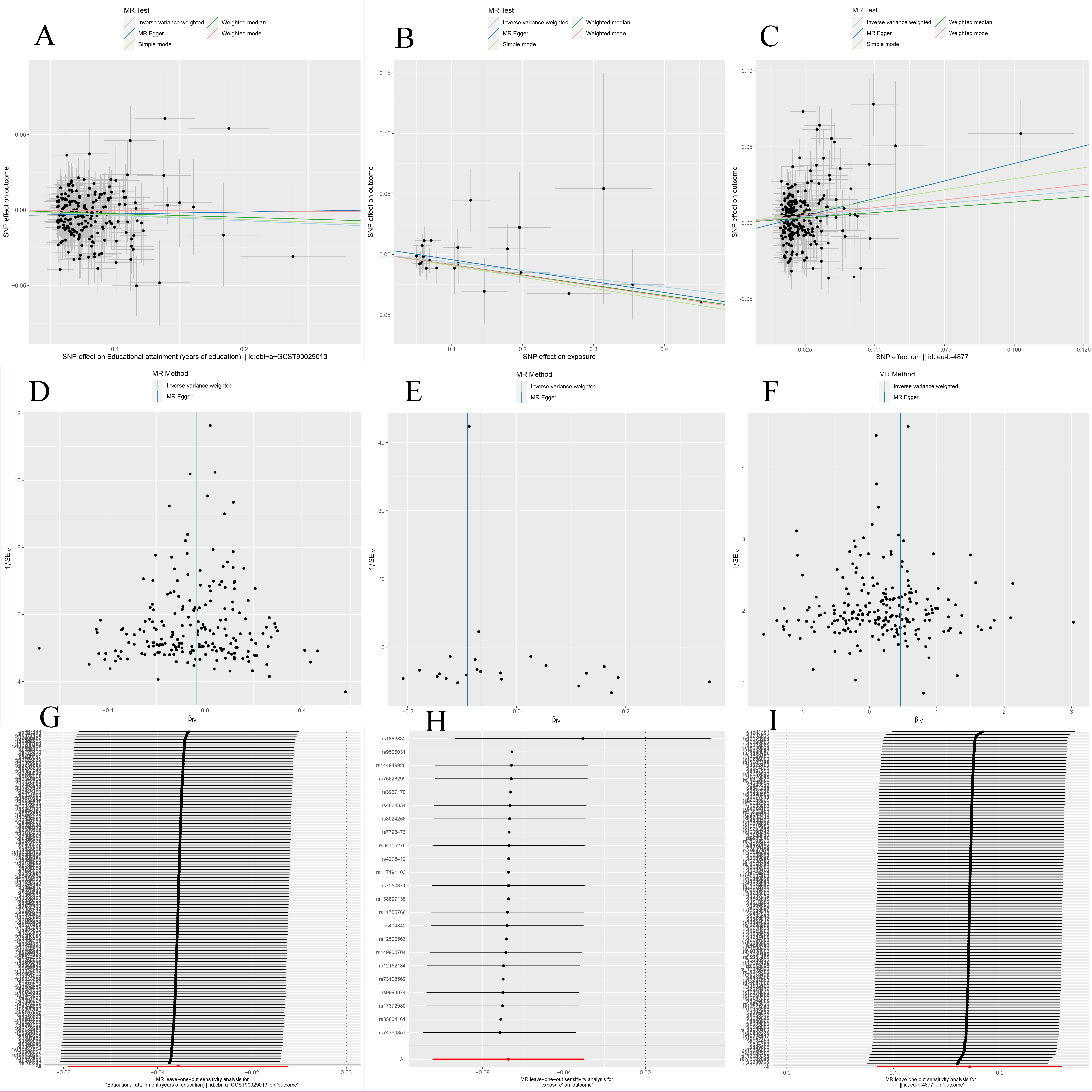


**Figure S2.** Scatter plots, funnel plots and “leave-one-out” results of genetic correlation between smoke initiation, C-X-C motif chemokine 11 levels, and atrial fibrillation by different MR analysis methods. A; D; G smoke initiation on C-X-C motif chemokine 11 levels. B; E; H C-X-C motif chemokine 11 levels. C; F; I smoke initiation on atrial fibrillation.


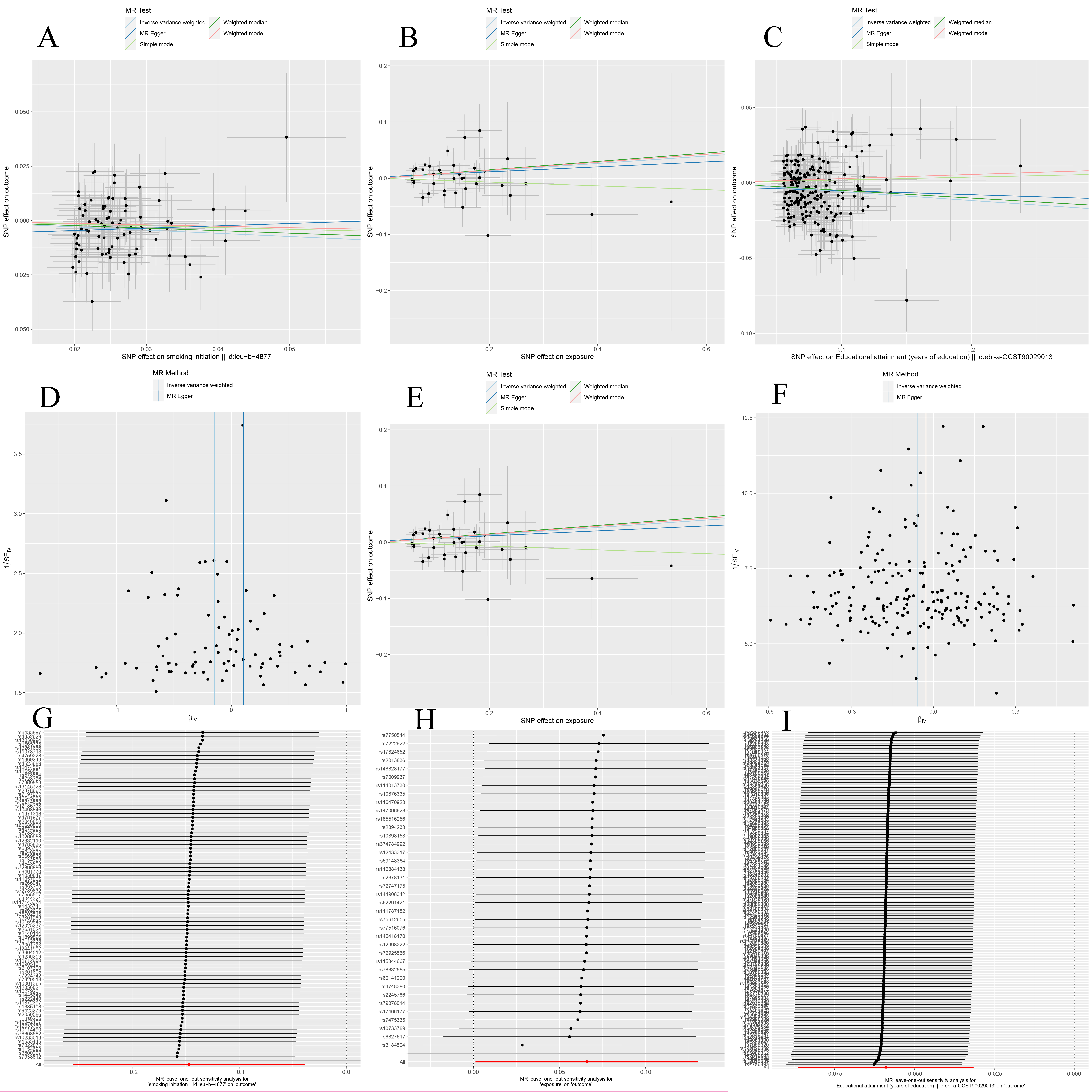


**Figure S3.** Scatter plots, funnel plots and “leave-one-out” results of genetic correlation between educational attainment (years of education), CD40L receptor levels, and atrial fibrillation by different MR analysis methods. A; D; G educational attainment (years of education) on CD40L receptor levels. B; E; H CD40L receptor levels on atrial fibrillation. C; F; I educational attainment (years of education) on atrial fibrillation.


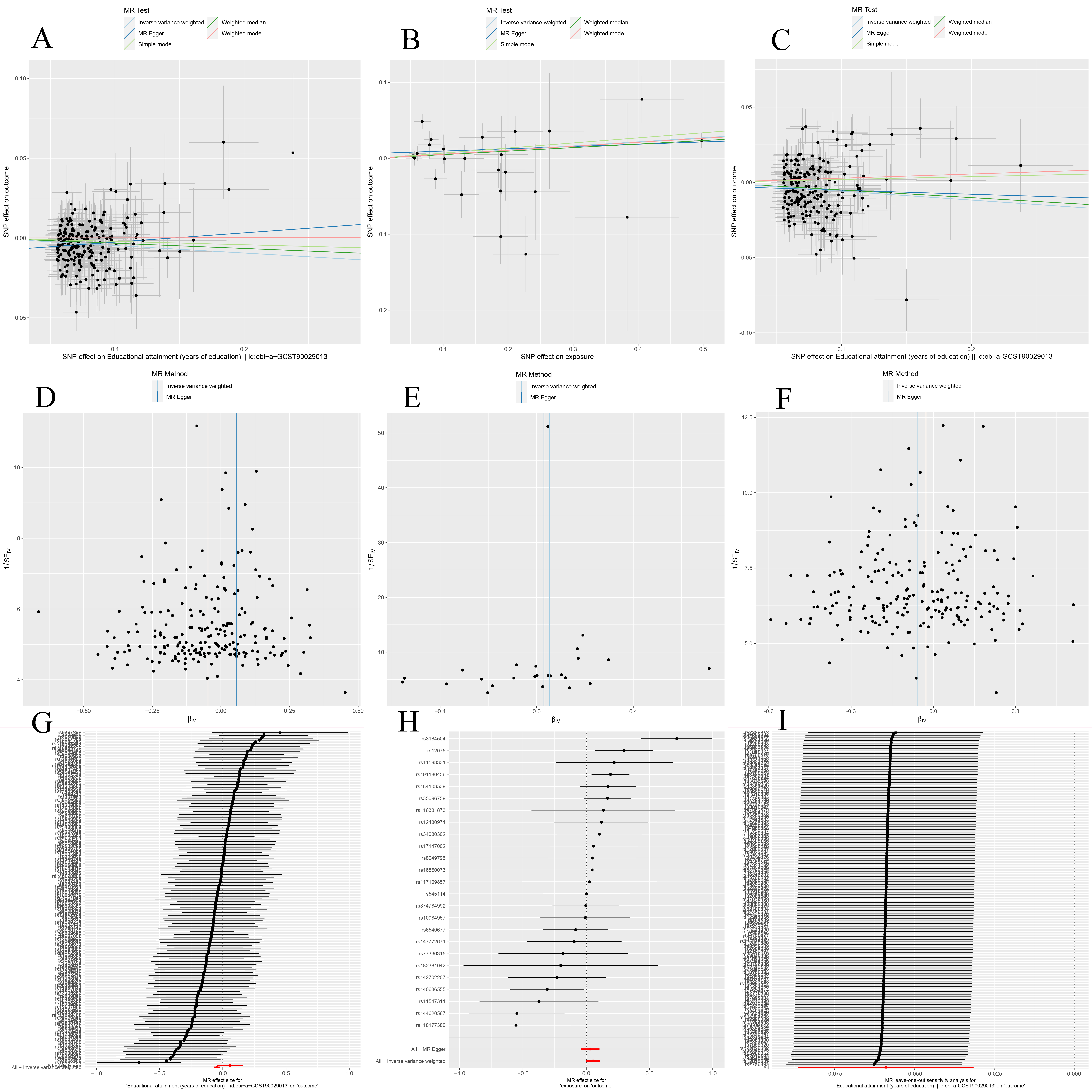


**Figure S4.** Scatter plots, funnel plots and “leave-one-out” results of genetic correlation between educational attainment (years of education), C-X-C motif chemokine 6 levels, and atrial fibrillation by different MR analysis methods. A; D; G educational attainment (years of education) on C-X-C motif chemokine 6 levels. B; E; H C-X-C motif chemokine 6 levels on atrial fibrillation. C; F; I educational attainment (years of education) on atrial fibrillation.
